# A Tale of Integration? The Migrant Wealth Gap in Austria

**DOI:** 10.1007/s10680-021-09604-1

**Published:** 2022-02-14

**Authors:** Mattias Muckenhuber, Miriam Rehm, Matthias Schnetzer

**Affiliations:** 1 Momentum Institute, Vienna, Austria; 2 Institute of Socio-Economics, University of Duisburg-Essen, Duisburg, Germany; 3 Austrian Federal Chamber of Labour, Vienna, Austria; 4 Department of Economics, Vienna University of Economics and Business, Vienna, Austria

**Keywords:** Migration, Wealth distribution, Wealth gap, Unconditional quantile regression, Integration, C31, D31, F22, G51, J15, J61

## Abstract

We investigate how previous generations of migrants and their children integrated into Austrian society, as measured by their wealth ownership. Using individual-level data from the Household Finance and Consumption Survey (HFCS), we document (1) a positive average migrant wealth gap between migrants and natives—that is, migrants owning less wealth than natives, especially in the upper half of the distribution, (2) substantial within-group inequality for migrants, and (3) evidence for catch-up, since second-generation migrants are much more similar to natives in terms of wealth and socio-economic characteristics than first-generation migrants. Using a RIF regression, we confirm an economically significant migrant wealth gap for first-generation migrants after controlling for socio-economic characteristics especially for the upper middle of the distribution, where housing wealth is a particularly relevant asset category. Second-generation migrants’ wealth gap is fully explained by our covariates in the middle of the distribution, whereas at the top where business wealth is more salient, their characteristics predict them to have *higher* wealth than natives. Decomposing the partial effects of covariates suggests that inheritances have the highest explanatory power for the migrant wealth gap of both first- and second-generation migrants, further buttressing the case for progressive integration in terms of wealth, while the composition of the migrant population, and in particular migrants’ heritage may continue to play a role in their wealth ownership.

## Introduction

Wealth is an indicator of integration: owning a home or a business heightens the sense of "belonging" in a host country. Wealth ownership conveys important functions, ranging from security, to the use value, to dynastic implications through bequests, and, ultimately, power. Wealth attainment thus adds to income and employment as key variable for the economic integration of migrants. However, opportunities to accumulate wealth are unequally distributed and chances to inherit assets differ by migration background (Gittleman and Wolff 2004; Semyonov and Lewin-Epstein 2013). These differences in socio-demographic characteristics between migrants and natives might result in a sizable migrant wealth gap—migrants owning less wealth than natives. Among the factors that affect wealth accumulation are direct effects like earnings capacity, saving behavior, rates of return, and wealth transfers. As data for these are rare and may be subject to reporting issues, the literature also relies on a host of indirect factors, among them age, education, marital status, employment status, and migration cohort. Successful integration implies that differences in these socio-demographic characteristics decline with the duration of stay or subsequent migration cohorts so that the wealth gap eventually diminishes. This paper thus asks the question: How do previous generations of migrants and their native-born descendants integrate into their destination society, as measured by their wealth ownership?

We examine the net wealth gap between natives and migrants at different percentiles of the net wealth distribution in Austria using the Household Finance and Consumption Survey (HFCS) 2014 provided by the European Central Bank. As a poster child for a historically multi-ethnic nation with strong anti-immigrant sentiments, the Austrian case corresponds to general migration patterns in continental Europe. The bulk of existing literature is based on data for the USA, and there is little empirical evidence for Europe. Applying recentered influence function (RIF) regressions on HFCS data, we decompose the migrant wealth gap into a composition effect, which is explained by differences in the distribution of socio-demographic characteristics, and a structure effect ascribed to differences in the returns to those characteristics. The previous literature on the net wealth gap either used OLS regressions or quantile regressions that depend on the order of the decomposition. Furthermore, most studies analyze wealth gaps on a household level, whereas our data enable us to investigate the migrant wealth gap at the individual level. We are in addition to studying the wealth gap at the individual level able to check the robustness of our results at the household level. We also distinguish various groups of migrants, such as first-generation migrants with a short and long time since arrival, and second-generation migrants.

We find that second-generation migrants—in contrast to first-generation migrants—are very similar to natives. For first-generation migrants, there is a substantial net wealth gap amounting to roughly €100,000 at the mean. This gap is larger for first-generation migrants with a shorter time since arrival ($\leqslant$20 years) and smaller for migrants who lived in Austria for more than 20 years. The gap is particularly large at the upper half of the distribution and only a small part can be explained by our control variables (age, gender, marital status, number of children, education, income, and inheritances). In particular, differences in the distribution of inheritances and age help explain the migrant wealth gap. Second-generation migrants, however, share much more similarities with natives in terms of net wealth but also with regard to most socio-demographic characteristics. Here, the net wealth gap is considerably smaller with merely €25,000 at the mean. What is striking is that the overall composition effect is negative, meaning that if second-generation migrants and natives had the same distribution of socio-demographic characteristics, the net wealth gap would be even larger. Contrary to the results for first-generation migrants, we find that inheritances have the largest negative effect on the gap for second-generation migrants.

## Theory and Literature Review

The accumulation of wealth may be described by the model

1 $$\begin{aligned} W_{t+1} = (1 + r_t)(W_t + Y_t - C_t \pm T_t), \end{aligned}$$

where wealth *W* at time $t+1$ consists of wealth at time *t* plus disposable income *Y* minus consumption *C* and wealth transfers *T*, multiplied by the rate of return *r*. All of these factors may differ between migrants and natives, and thus help explain why a migrant wealth gap exists, i.e., why migrants may hold lower wealth than natives (for the gender wealth gap, compare Schneebaum et al. 2018).

First, there is ample evidence of a migrant wage gap, implying that income *Y* of migrants is lower than that of natives (Lehmer and Ludsteck 2015; Hofer et al. 2017; Beyer 2019). These studies show by means of decomposition analyses that part of the wage gap can be ascribed to observable differences between migrants and natives mainly in educational attainment and work experience (Nielsen et al. 2004), while part of the gap is due to unobserved characteristics or plain discrimination. Another argument is that migrants may work in occupations below their formal qualification as they lack host country-specific human capital (Ingwersen and Thomsen 2021). Hence, earnings are low at the time of arrival but integration, such as acquiring knowledge on language, customs, and labor market specifics, likely leads to the diminution of the initial wage gap. Wages of naturalized migrants thus tend to be much closer to those of natives compared to other migrants, as domestic citizenship might be favored in the labor market and more integrated migrants are also more likely to be naturalized (Aldashev et al. 2012).

Second, according to the life-cycle hypothesis, wealth typically follows an inverted u-shaped pattern over the life span; it is built up during the economically active life phase, and consumed after retirement. If migrants are on average younger than natives, then age explains why migrants have lower wealth than natives (because they are at a different - lower - point in their lifecycle consumption out of income). Controlling for age should then capture that consumption *C* may differ between migrants and natives. Furthermore, the size of the household indicates on the one hand the ability to accumulate wealth due to a larger number of economically active members, and on the other hand larger housing requirements and consumption expenses. Controlling for differences in household size between migrants and natives should thus account for differing consumption patterns.

Third, transfers play a role in wealth accumulation, in particular inheritances and remittances. If wealth levels differ by country, then average inheritances will also vary by country of origin, leading to differences between migrants and natives. If migrants are not representative of the population in their country of origin—for instance because poorer households are more likely to diversify their income sources by sending one member as migrant—, then this may compound the differences in average inheritances. Furthermore, if refugees make up a substantial part of the migrant population, this may also lead to differences in inheritances in comparison to natives, since their family wealth in the country of origin may have been destroyed. Lastly, remittances are regular wealth transfers from migrant workers to their countries of origin and thus impact migrants’ wealth negatively if they involve a transfer of ownership (Garip 2014; Kangmennaang et al. 2018).

Finally, migrants may face a lower rate of return compared to natives for a number of possible reasons. For instance, migrants may have lower (possibly country-specific) financial literacy. Their asset composition may also differ from the asset composition of natives, leading to differential returns since bank deposits, for instance, yield lower returns than equity (Ederer et al. 2020). Furthermore, migrants may be less likely to employ professional portfolio management or have less access to formal and informal networks with investment information, and thus be less able to reap advantages from high-yield investment opportunities (Piketty 2014; Fagereng et al. 2020).

The difference between migrants and natives in all these respects is mitigated by integration: the time since arrival provides a temporal axis for the speed with which migrants’ wealth can be expected to approximate natives’ wealth. *Within* a generation, this may be due to higher income as a result of better labor market access. This may for instance be the case if the legal status (e.g., work permits, nostrification of educational degrees), language acquisition, or employment opportunities improve over time. *Across* generations, the integration of migrants’ children into the Austrian education system as well as intergenerational transfers may mitigate the migrant wealth gap.

There is a growing empirical literature on the migrant wealth gap, which has investigated Canada (Shamsuddin and DeVortez 1998; Zhang 2003), the USA (Hao 2004; Gittleman and Wolff 2004; Cobb-Clark and Hildebrand 2006; Bauer et al. 2011; McKernan et al. 2014; Hamilton and Darity 2017), New Zealand (Gibson et al. 2007), Australia (Doiron and Guttmann 2009; Bauer et al. 2011), Germany (Bauer et al. 2011), and Israel (Lewin-Epstein and Semyonov 2013). Regarding components of net wealth, housing is the most well-studied (Coulson 1999; Borjas 2002). All of these papers find a (positive) migrant wealth gap, that is, migrants hold less wealth than natives at least at some point of the distribution.

Regarding methods, whereas some papers use OLS (Shamsuddin and DeVortez 1998; Lewin-Epstein and Semyonov 2013) or Tobit regressions (Hao 2004), decomposition of quantile regressions has become the standard approach. The literature on migrant wealth gaps typically uses approaches introduced by DiNardo et al. (1996) (Zhang 2003; Cobb-Clark and Hildebrand 2006; Gibson et al. 2007; Bauer et al. 2011) or Machado and Mata (2005) (Doiron and Guttmann 2009). While these approaches are an important advance over the analysis of averages in OLS, they only permit investigating the conditional distribution of wealth, and their results are path dependent (that is, the results depend on the order of the decomposition). In this paper, we apply recentered influence function (RIF) regressions (Firpo et al. 2018) to address these shortcomings. RIF regressions have the advantage of estimating effects across the unconditional distribution of net wealth, and being path independent.

The literature investigates a number of the theoretical explanatory factors identified above. Since data for direct effects on wealth accumulation such as earnings capacity, saving behavior, rates of return, and wealth transfers (especially remittances) are rare and, if available, often plagued by reporting issues, the literature relies on a host of indirect factors. The most commonly studied are demographic variables (which typically include age, children, and sometimes marital status), economic variables (especially income, but also education), as well as migration-related variables (in particular duration of stay and country of origin).

A number of studies find integration in the sense that the migrant wealth gap closes with the duration of stay or subsequent migration cohorts, especially for Canada. Shamsuddin and DeVortez (1998) and Hao (2004) suggest that migrants to Canada catch up with natives within 15 to 22 years. Zhang (2003) also indicates catch-up in Canada. In contrast, Doiron and Guttmann (2009) do not find that the migrant wealth gap closes over time in Australia, and Borjas (2002) provides descriptive evidence of declining levels of assimilation into home ownership by migrants in the USA.

## Migration to Austria

Migration has always played an important role in Europe, and Austria is a poster child for the temporary guest-worker policies in Austria, Germany, and Switzerland. The complex migration flows in Europe’s post-war history can be simplified into a sequence of labor migration up to the 1970s, family reunification in the 1970s, and refugee migration starting in the 1980s (Hansen 2003). For Europe, the literature distinguishes between two general types of labor migration: (post-)colonial migration particularly in the UK, France, the Netherlands, and Belgium; and temporary guest-worker policies for instance in Austria, Switzerland, and Germany (Hansen 2003). In the early 1970s, however, most European countries ended primary migration and gradually increased family reunifications. From 1980 and particularly after 1989, refugee migration gained momentum in Europe as violent conflicts, falling transportation costs and the fall of the Berlin Wall reinforced the need and motivation for many people to resettle.

Austria has followed these European migration trends against the backdrop of its specific history and geographic location. The country has a longstanding tradition of migration dating back to the Habsburg Empire, which encompassed 14 nationalities, nine official languages, and five recognized religions. This era was mainly characterized by internal migration within the empire, mostly linked to specific labor markets in other regions (Steidl 2017). After World War II, however, Austria exhibited migration patterns very similar to those of other European countries, in particular Germany. In the 1960s, it actively attracted a workforce mainly from Turkey and former Yugoslavia due to domestic labor shortages in the prosperous post-war period. Temporary recruiting often turned into permanent employment, and family reunions offset falling immigration numbers following a recruitment ban in the 1970s. Political reasons were another push factor for migration to Austria due to its proximity to the former Soviet Union and to the violent disintegration of former Yugoslavia. While political incidents characterized migration flows beginning with the 1990s, there are also historic cases of refugee movements to post-war Austria, like 1956 from Hungary, 1968 from Czechoslovakia or 1981 from Poland (Rupnow 2017).

Today, almost a quarter of the Austrian population either migrated themselves or have at least one parent who migrated (Statistics Austria 2014). OECD (2020) data show that the share of foreign-born population in Austria is similar to Germany’s at about 19% versus 16%. This is well below Switzerland and Australia with about 30% migrants, but higher than large countries with a colonial history like France, Spain, the United Kingdom, and the USA, whose migrant shares are below 15%.

Despite country-specific idiosyncrasies, continental Europe shares general migration patterns and histories to a certain degree. The strong and longstanding tradition of migration in Austria makes it an interesting case for the analysis of economic integration of migrants in terms of wealth. Given that individuals mainly moved to Austria due to economic or political reasons and potentially arrived with only little resources, this historical analysis leads us to expect a wealth gap between natives and first-generation migrants. This gap should decrease with the duration of stay and diminish for second-generation migrants born in Austria.

## Data

Our analysis of the net wealth gap between natives and migrants is based on data of the Household Finance and Consumption Survey (HFCS) 2014 (European Central Bank 2014). The HFCS is a representative survey that collects harmonized information on households’ finances in euro area countries. It contains complex survey weights, replicate weights, and multiple imputations. All calculations presented here are weighted and take the five available multiple imputations into account by using Rubin’s rule (Rubin 1987). The HFCS provides detailed data on households’ net wealth and its components, as well as a plethora of demographic and socio-economic characteristics. The Austrian data set also includes non-core questions on the distribution of net wealth between household members, as well as on the migration background of each household member, which allows us to differentiate first- and second-generation migrants, and to split first-generation migrants into those with a shorter and those with a longer time since arrival. All variables used here are self-reported in the HFCS.

The Austrian HFCS sample covers a total of 6,189 individuals. Excluding multi-generation households (which would complicate our relationship status categorization) from our sample leaves us with 4,170 observations.

The main variable of interest is net wealth, which is defined as the sum of a household’s real and financial assets minus its debt. Real assets consist of real estate wealth, vehicles, valuables and self-employment businesses. Financial assets are deposits, non-self-employment businesses, shares, bonds, mutual funds, managed accounts, other financial assets, voluntary pensions, and money owed to the household. Deducted from these are mortgages and other debt. A non-core variable asks respondents to allocate the net wealth among all household members. The resulting person-level net wealth is our dependent variable.

We determine migration background based on two items in the Austrian HFCS questionnaire: The survey asks whether household members (1) migrated themselves or (2) at least one of their parents did. If the response is affirmative to the first question, then the individual is coded as first-generation migrant. If a parent migrated, then we define the individual as second-generation migrant. Furthermore, we re-assign migrants who moved to Austria before the age of six years (i.e., before school age) from first- to second-generation migrants, since they were arguably socialized in Austria. We also exploit the year in which migrants first moved to Austria to further divide first-generation migrants into two groups, those with a short and those with a long time since arrival with a cutoff at 20 years. Finally, our data contain information on the region of origin in three categories: other EU member states, other Europe, and rest of the world. Due to our limited sample size, we use time since arrival and region of origin only to investigate the structure of our migrant population descriptively. Of our total 4,170 observations, 337 (8.1%) observations are second-generation migrants, 417 (10%) are first-generation migrants, and of these, 206 (49.4%) have been in Austria for 20 years or less and conversely, 211 (50.6%) arrived longer than 20 years ago.

In our multivariate results in Sect. 6, we follow the literature in controlling for wealth accumulation using demographic factors (age, gender, relationship status, the presence of children under 25 years of age), factors determining income-earning capacity (education level, gross income), and wealth transfers (the receipt of above-average inheritances) into our analysis. All control variables are coded as dummies.^1^ The cutoff for age is its median, 53 years.^2^ Relationship status is defined as single or living with a partner in the household. Educational attainment is measured using the ISCED-97 classification, and it is classified as high starting with a high-school degree (ISCED level 3). Gross income consists of employee and self-employment income, capital income, and transfers. We divide the latter two, which are only recorded at the household level, according to the person-level ownership of net wealth. The threshold for our high income dummy variable is its median (€23,034). The value of inheritances, which the HFCS contains in the year in which they were received, is inflated using the CPI.^3^ Since our data only contain inheritances at the household level, we assume that they are distributed like net wealth. High inheritances are defined as those larger than median net wealth of individuals in the HFCS (€48,863).^4^

**Table 1:** Summary statistics *Source*: own calculations, HFCS (2014)

		Natives	Migrants			$1{\mathrm{st}}$ Gen migrants	
			Total	$1{\mathrm{st}}$ Gen	$2{\mathrm{nd}}$ Gen	Short	Long
Net wealth	Mean	165,730	98,007	63,001	139,775	39,598	86,317
	SD	(24,851)	(32,071)	(8850)	(68,190)	(8042)	(16,055)
	Median	59,001	15,931	9917	32,763	4935	20,196
Age: old	%	51.9	43.3	36.4	51.5	15.0	57.7
Gender: male	%	46.0	46.0	46.1	45.8	49.7	42.5
Marital St.: single	%	30.7	28.9	23.4	35.4	20.4	26.5
No children	%	70.2	61.4	53.5	70.8	44.6	62.4
Education: high	%	84.3	81.0	79.0	83.4	81.2	76.8
Income: high	%	50.7	46.6	42.6	51.4	41.9	43.2
Inheritance: high	%	18.6	14.1	8.6	20.6	6.7	10.5
N		3416	754	417	337	206	211

This table shows mean, standard deviation (SD), and median of net wealth, as well as the shares in controls of natives, first-, and second-generation migrants. First-generation migrants are further distinguished into short ($\leqslant$20 years) and long (>20 years) time since arrival. For age, the cutoff is the sample median of 53 years, for education ISCED level 3 or higher, for income its median, and for inheritance the median of net wealth

Table 1 contains summary statistics of our data for natives and migrants, with the latter split into first- and second-generation migrants, and first-generation migrants differentiated further into those with a short or long time since migration. It shows two stylized facts regarding the wealth distribution: First, natives have substantially higher net wealth both on average and at the median compared to migrants (first and second column in Table 1). This indicates the migrant wealth gap typically found in the literature: Natives own more wealth than migrants.

Second, the differences between first- and second-generation migrants (columns three and four in Table 1) are larger than those between natives and migrants in general. While the mean net wealth of second-generation migrants at about €140,000 is almost as high as the mean net wealth of natives (about €166,000), net wealth of first-generation migrants is only around €63,000. First-generation migrants, in turn, are closer to the migrant population’s average net wealth (about €98,000) if they have resided in Austria for a long time at about €86,000, and have very low mean net wealth of about €40,000 if they arrived a short time ago. However, it should be noted that the sample size becomes very low with this fine-grained differentiation.

These stark differences between first- and second-generation migrants, and first-generation migrants with a short and long time since arrival, carry over into the distributional analysis. While the distribution of net wealth is highly right-skewed for both natives and migrants, inequality as measured by the ratio of mean to median net wealth indicates that net wealth is distributed more unequally within migrants than within natives: It amounts to almost three for natives and roughly six for migrants. Furthermore, among migrants, the distribution is more unequal for first-generation migrants than for second-generation migrants at mean-median net wealth ratios of 6.4 and 4.3, respectively, and among first-generation migrants for those with a short (8) and long (4.3) time since migration.

Finally, the similarity between second-generation migrants and natives is reinforced by their characteristics, which we show as population shares for all control dummy variables in Table 1. In every dimension except gender—couples versus singles, children^5^, age, level of education, and the level of income and inheritances—, second-generation migrants are much more similar to natives than first-generation migrants. The data on the control variables also show that first-generation migrants with a long time since migration are generally more similar to the total migrant population than those with a short time since arrival, with few exceptions: Table 1 shows that first-generation migrants who arrived a short time ago are disproportionately young and have higher shares of males, high educational attainment and children than first-generation migrants with an earlier time of arrival. Consequently, income levels do not differ markedly between first-generation migrants with a short and a long time since arrival.

**Fig. 1:**
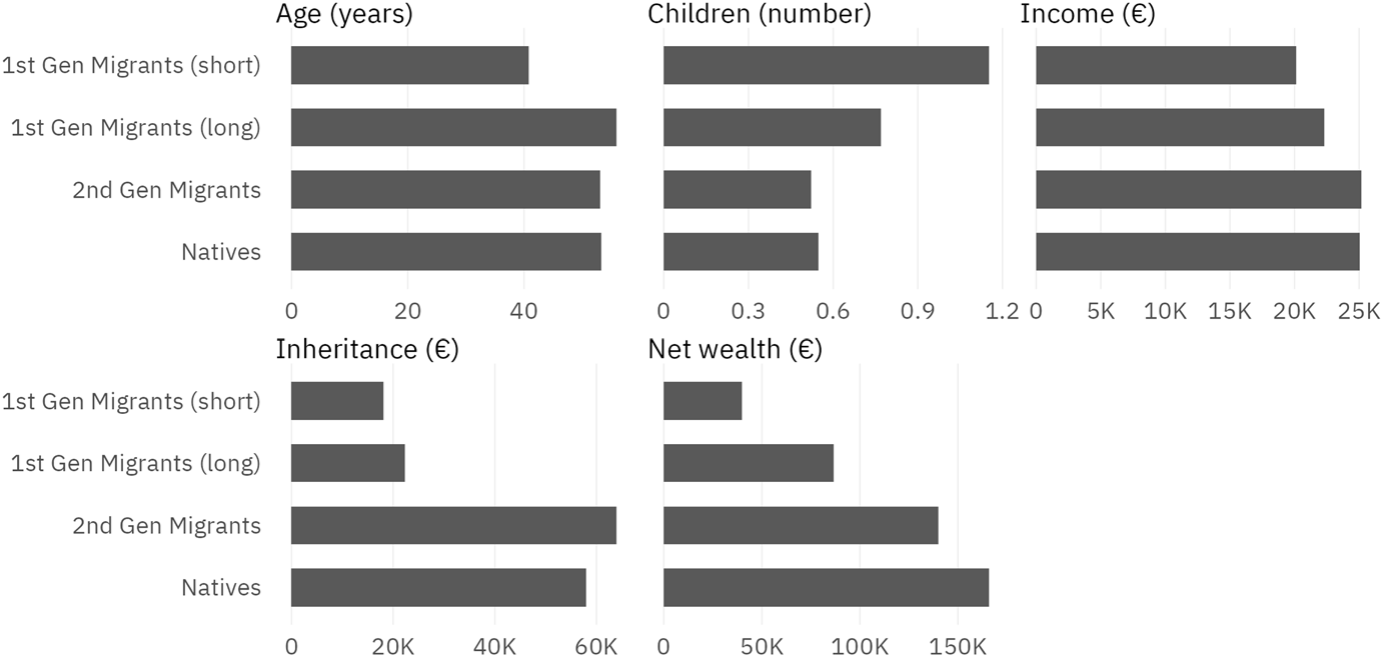
Average characteristics by migration status. *Source*: own calculations, HFCS (2014). Note: This figure shows the unconditional means for first-generation migrants with a short ($\leqslant$20 years) and long (>20 years) time since arrival, second-generation migrants, and natives

In order to explore these differences between our population groups further, Fig. 1 shows the averages of our numerical variables for first-generation migrants (by time since arrival), second-generation migrants, and natives. It suggests that first-generation migrants are younger, have more children, lower income and especially inheritances, and have less net wealth than second-generation migrants and natives. There appears to be some further differentiation of first-generation migrants by time since arrival along these same lines; however, it should be noted that the number of observations for these groups is so low that these point estimates are highly uncertain. Second-generation migrants, in contrast, are very similar to natives in their economic and demographic characteristics - with the exception of somewhat higher average inheritances and lower net wealth.

Finally, we investigate the region of origin of migrants in more detail, which Fig. 5 in the Appendix shows for second-generation migrants and first-generation migrants (again by time since arrival). Having been born outside the EU appears to be negatively correlated with an individual’s position in the net wealth distribution; there is an inverted u-shaped relationship between "Rest of the European Union" as a region of origin and the level of net wealth; and the likelihood for persons to originate from the Euro area seems to rise with the net wealth distribution. Here, too, the number of observations is very low especially for the two groups of first-generation migrants, so that these findings should be interpreted very cautiously.

**Fig. 2:**
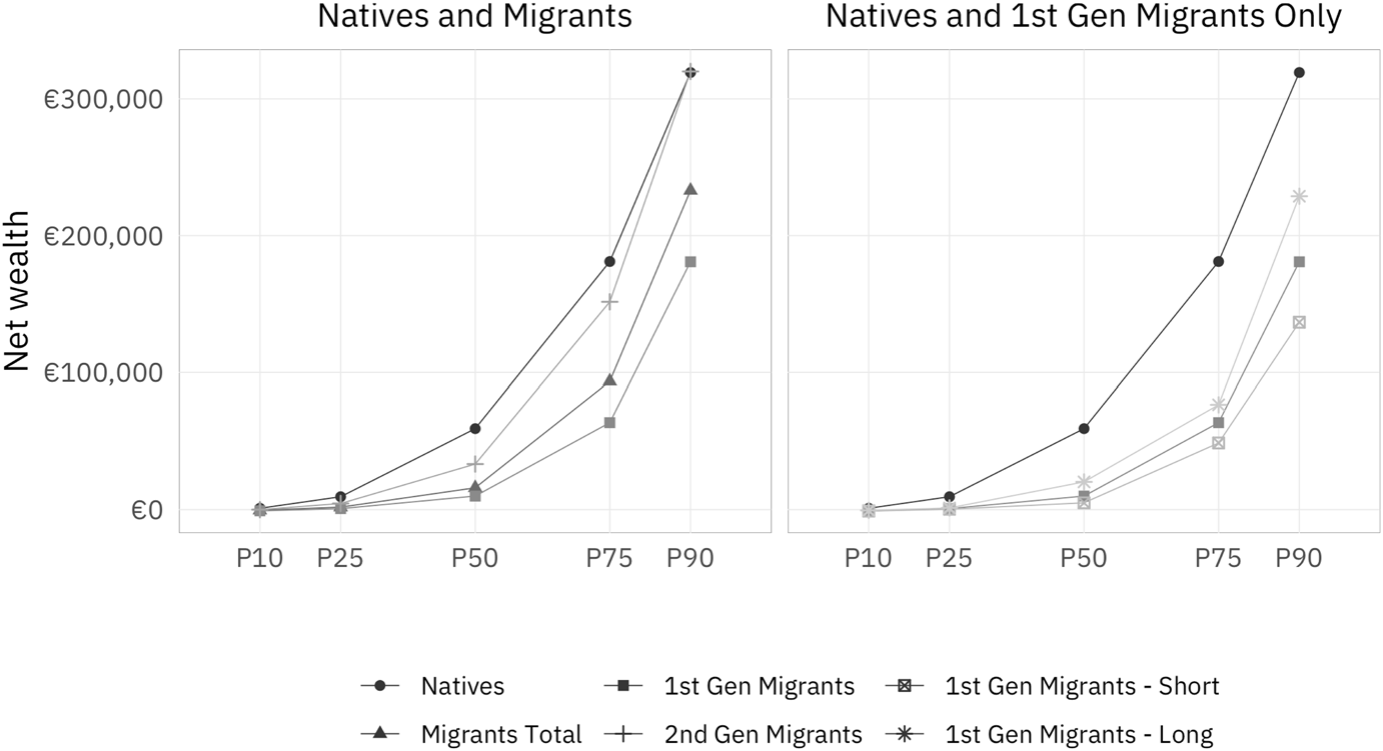
Net wealth by migration status at selected percentiles of wealth distribution. *Source*: own calculations, HFCS (2014). Note: The left-hand side panel of this figure shows net wealth for natives and for all migrants, as well as first- and second-generation migrants at selected percentiles of net wealth distribution. The right-hand side panel shows the same information for first-generation migrants, as well as first-generation migrants with a short ($\leqslant$20 years) and long (>20 years) time since arrival

Next, we turn to the raw migrant wealth gap, that is, the difference between natives’ and migrants’ net wealth. Figure 2 shows net wealth by migration status at selected percentiles of the net wealth distribution. The left-hand side panel shows that there is a substantial wealth gap between migrants (triangles) and natives (circles). However, the lines in lighter gray differentiating first-generation migrants (squares) and second-generation migrants (plus signs) indicate that the net wealth curve of second-generation migrants closely tracks that of natives. The overall migrant wealth gap is roughly €43,000 at the median. It rises to almost €87,000 at the $75^{\mathrm{th}}$ percentile, before stabilizing at the top of the distribution (approximately €86,000 at the $90^{\mathrm{th}}$ percentile.)

The right-hand side panel shows that the migrant wealth gap between natives and first-generation migrants decomposes into a smaller gap for first-generation migrants with a long time since arrival, and a somewhat larger gap for those with a short time since arrival. They reach €90,000 and €183,000, respectively, at the $90^{\mathrm{th}}$ percentile.

To conclude, the descriptive evidence indicates substantial differences between migrants and natives with regard to net wealth, especially for first-generation migrants and—with caution—in particular first-generation migrants with a more recent time of arrival. In contrast, second-generation migrants are much more similar to natives than first-generation migrants. Furthermore, wealth is distributed more unequally within migrants than within natives, and more unequally within first-generation migrants than within second-generation migrants. Finally, the descriptive data also show that these differences between first- and second-generation migrants largely carry over to socio-economic characteristics. In particular, first-generation migrants are younger, have more children, lower income, and especially lower inheritances than both second-generation migrants and natives. While they should be interpreted with caution, the descriptive data also suggest that first-generation migrants with a short time since arrival are more likely to be male, younger, and less likely to be from the EU than first-generation migrants on average.

The descriptive bi-variate evidence presented in this section suggests a strong catch-up of previous cohorts of first-generation migrants, i.e., second-generation migrants’ parent generation bequeathing them inheritances even higher than natives’ parents, which may lead to a partly unexplained migrant wealth gap for second-generation migrants. These findings are consistent with either a progressive economic integration of first-generation migrants, or with a cohort effect, which we are unfortunately unable to disentangle with our data.

However, as Fig. 2 shows for net wealth, the averages presented in Fig. 1 hide economically significant differences across the distribution. These differences are far from uniform, so an analysis of averages is likely to be misleading. Whether the descriptive evidence at the mean from this section carries over to the entire distribution of net wealth, which factors contribute to explaining the unconditional wealth differences between natives and both first- and second-generation migrants at different points of the distribution, and whether the migrant wealth gap remains unexplained in a multivariate analysis, are the questions that we aim to answer in the next sections.

## Methodology

We use recentered influence function (RIF) regressions (Firpo et al. 2018) to decompose the net wealth gap between natives and first as well as between natives and second-generation migrants into contributions of socio-demographic factors. First, we obtain counterfactual distributions of migrants *as if* they had the (observable) characteristics of natives. Using the observable characteristics of natives and migrants in a logistic regression allows us to obtain inverse probability weights. This way, analogous to Kitagawa–Oaxaca–Blinder decompositions (Kitagawa 1955; Oaxaca 1973; Blinder 1973), we are able to assess the part of the migrant wealth gap that can be explained by differences in the composition of individual groups and the part that remains unexplained. The RIF regression then uses the recentered influence function as the dependent variable in an OLS specification to estimate the impact of the control variables at specific quantiles of the migrant wealth gap. Thus, we are able to quantify the explanatory power of each variable for the migrant wealth gap.

The RIF is defined as:

2 $$\begin{aligned} RIF(y; q_{\tau }, F) = q_{\tau } + IF(y; q_{\tau }, F) = q_{\tau } + \frac{\tau - \mathbbm {1}\{y \le q_{\tau } \}}{f_{Y}(q_{\tau })}. \end{aligned}$$

where $\tau$ is the quantile of interest, $q_\tau$ is the value of the quantile of interest, and $\mathbbm {1}$ is an indicator which is 1 if an individual’s net wealth *y* is below the value of the quantile of interest $q_{\tau }$^6^, and 0 otherwise. $f_{Y}(q_{\tau })$ is the kernel density estimator at the value of the quantile of interest $q_{\tau }$ using a Gaussian kernel. A neat characteristic of the influence function (IF) is that its expected value is equal to zero. Since the RIF consists simply of the quantile of interest added to the IF, we can make use of the fact that its expected value is equal to the quantile itself (e.g. the expected value of the RIF of the median is equal to the median).

Next, we regress the RIF on our covariate vector *X* for each group, i.e., natives, migrants, and the counterfactual, which is migrants reweighted to have the same distribution of *X* as natives. The composition effect, which measures the explained differences and thus compares migrants to the counterfactual, is then:

3 $$\begin{aligned} \Delta ^{\tau }_{X} = \sum ^{K}_{k = 1}{(\mathbb {E}[X^{k}|T = 1] - \mathbb {E}[X^{k}|T = 0])\gamma ^{\tau }_{0,k}} + R^{\tau }. \end{aligned}$$

which is the sum of differences between the expected values of the covariate vector *X* for the "treatment" group 1 (here, migrants with counterfactual characteristics $T=1$) and the control group 0 (here, migrants with ‘actual’ characteristics $T=0$). This is multiplied with the ‘returns’ on the covariates of migrants, that is, the coefficients recovered from regressing the RIF on the covariates for group 0, $\gamma _{0,k}$. $R^{\tau }$ is an approximation error.

## Results

**Fig. 3:**
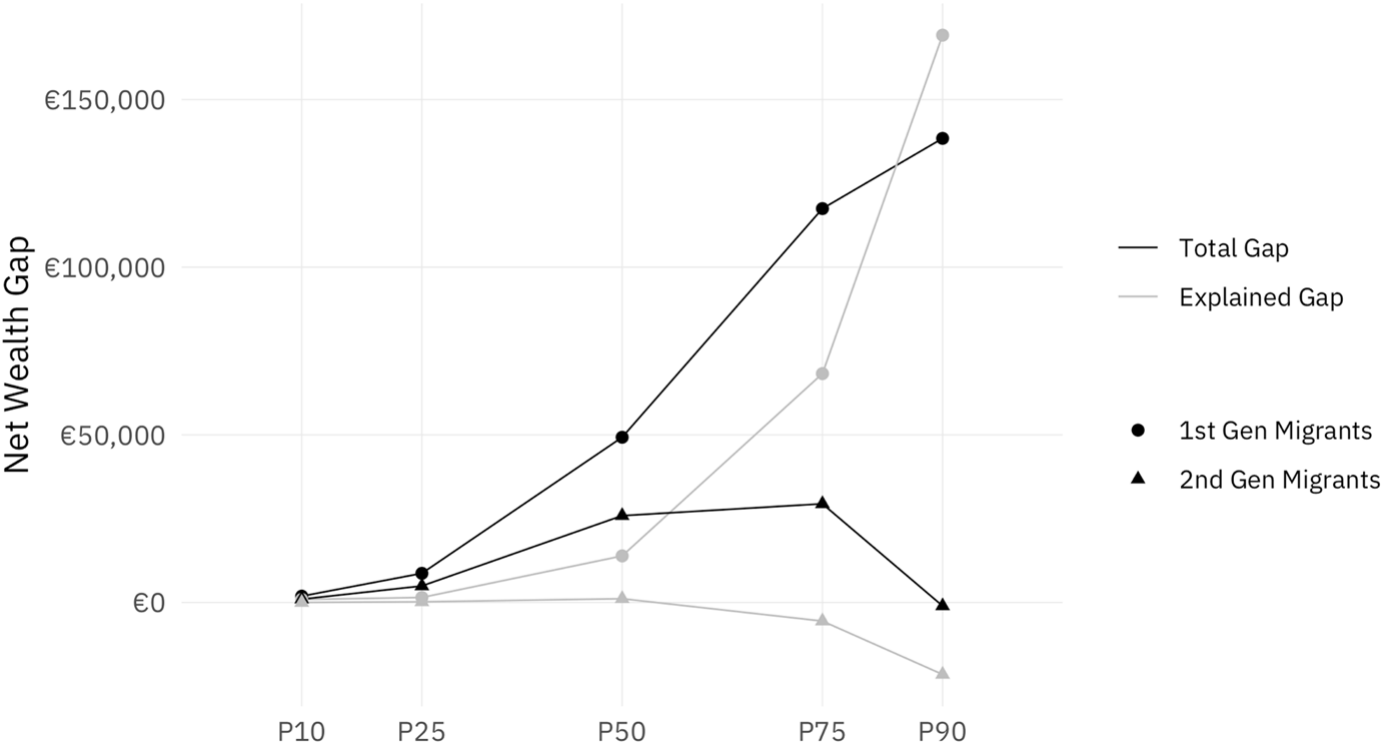
Decomposition of the migrant net wealth gap in Austria. *Source*: own calculations, HFCS (2014). Note: This figure shows the absolute raw gap (black lines) and the explained gap (gray lines) in net wealth between first- (circles) and second (triangles)-generation migrants and natives

We first present results for the overall net wealth gap with respect to migration status and then turn to the partial effects of socio-demographic characteristics. Figure 3 shows the absolute raw gap (black lines) and the explained gap (composition effect, gray lines) in net wealth between first- (circles) and second (triangles)-generation migrants and natives at the 10th, 25th, 50th, 75th and 90th percentile of net wealth distribution. The difference between the explained gap and the raw gap is the part of the migrant wealth gap that can *not* be attributed to the explanatory variables.

We observe a small but positive net wealth gap in the raw data between second-generation migrants and natives across the distribution, topping at almost €30,000 at the 75th percentile. However, the negative explained gap at the upper half of the wealth distribution indicates that we would expect second-generation migrants to own *more* wealth than natives based on their socio-demographic characteristics.

For first-generation migrants, on the other hand, there is a large absolute gap in net wealth compared to natives especially in the upper half of the distribution.^7^ While only a small part of this gap can be explained by differences in individual characteristics at the median, the composition effect rises steeply at the top of the distribution. In fact, at the top socio-demographic characteristics explain *more* than the large raw wealth gap of €138,000.

Tables 2 and 3 in the Appendix show the detailed RIF regression results for migrants and natives at selected percentiles of net wealth distribution. The directions of the statistically significant estimated effects are consistent for natives, first- and second-generation migrants, and fit well with the literature in almost all cases: Being single and not having children (apart from first-generation migrants) have a negative effect, while higher age, education, income, and inheritance all have a positive effect. The exception is gender, where being female in most cases has a positive effect on wealth.^8^ Figure 6 in the Appendix provides a graphic illustration of the regression results.

**Fig. 4:**
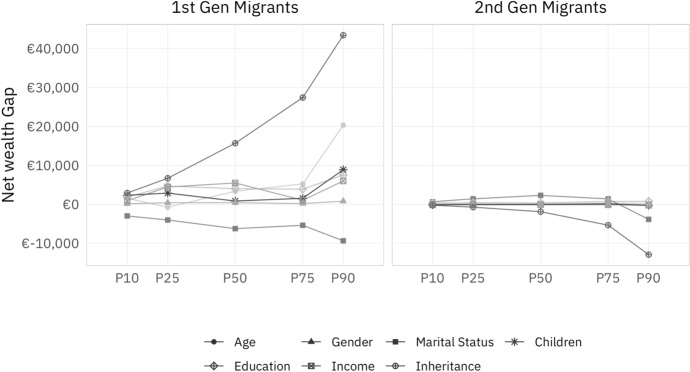
Partial effects of controls for migrants. *Source*: own calculations, HFCS (2014). Note: This figure shows the partial effects of the control variables age, gender, marital status, children, education, income, and inheritance in explaining the migrant wealth gap between natives and first- and second-generation migrants across the unconditional wealth distribution

Figure 4 allocates the explained part of the migrant wealth gap, as shown in Fig. 3, to contributions of individual explanatory variables. As discussed above, the composition effect is calculated as the differences in the means of the covariates of counterfactuals and migrants, multiplied by the RIF regression coefficients for migrants. This approach is able to reveal counteracting effects of individual variables that may be hidden in the aggregate perspective of the explained gap shown in Fig. 3.

For first-generation migrants, apart from the demographic factor age, Fig. 4 shows that it is mainly the socio-economic variables inheritances, education, and income, which contribute towards explaining the wealth gap to natives. In particular, the receipt of inheritances explains more than €40,000 of the migrant wealth gap at the top of the unconditional wealth distribution. As shown in Table 1, there are substantial differences in these variables between natives and first-generation migrants. Some 19% of natives receive inheritances larger than median net wealth, but only about 9% of first-generation migrants. Moreover, natives are substantially older with 52% older than the average, compared to about 36% for first-generation migrants and more likely not to have children living in the household (70% versus 54% for first-generation migrants). In contrast, marital status *decreases* the migrant wealth gap (or, equivalently, raises the wealth gap to be explained), as first-generation migrants are more likely to live in a relationship (77% versus 69% for natives). While the effect size tends to increase with the unconditional wealth distribution, the factors explaining a positive migrant wealth gap (that is, natives owning more wealth than first-generation migrants) are larger in absolute terms. This leads to the positive net contribution of our control variables towards explaining the wealth gap for first-generation migrants documented in Fig. 3.

For second-generation migrants, the picture is very different. As indicated in Fig. 3, the migrant wealth gap is much smaller for second-generation migrants, and the composition effect suggests that migrants’ wealth would be higher than natives’ if they had the same socio-demographic characteristics. The partial effects of the explanatory variables shown in Fig. 4 are thus much smaller in size: Age, gender, children, education, and income are hardly discernible. Only inheritances (and marital status in the $90{\mathrm{th}}$ percentile) have a negative effect. That is, given the higher inheritances among second-generation migrants compared to natives, we would expect migrants to have higher wealth than natives. The explanatory power of inheritances rises with the unconditional net wealth distribution, which largely drives the explained gap shown in Fig. 3.

**Fig. 5:**
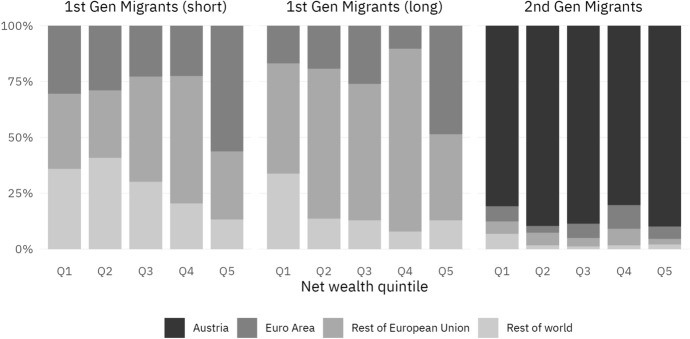
Country of birth of migrants by net wealth quintiles. *Source*: own calculations, HFCS (2014). Note: This figure shows the composition of migrants by country of birth and net wealth quintiles. First-generation migrants are distinguished between a short ($\leqslant$20 years) and long (>20 years) time since arrival

**Fig. 6:**
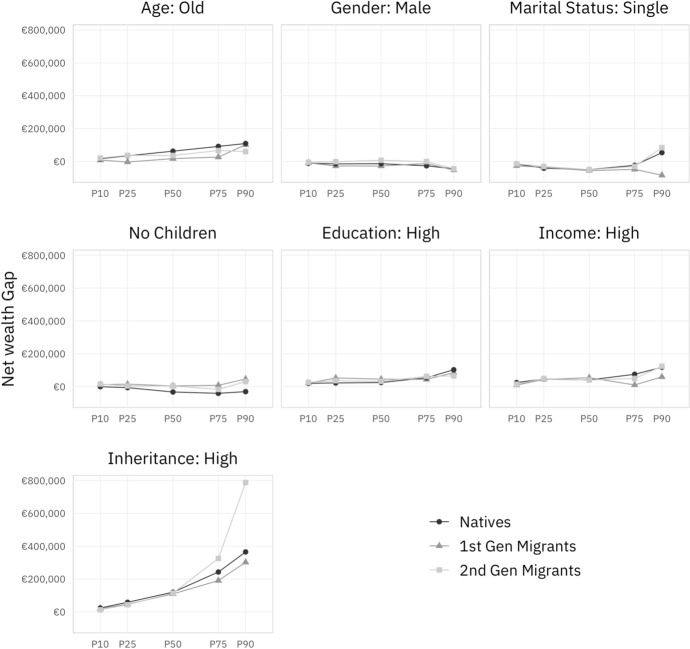
Coefficients for natives, $1{\mathrm{st}}-$ and $2{\mathrm{nd}}$-generation migrants. *Source*: own calculations, HFCS (2014). Note: This figure shows the coefficients of the control variables education, age, gender, marital status, children, income, and inheritance in explaining the migrant wealth gap across the unconditional wealth distribution

Naturally, these findings should be interpreted with caution. The sizeable unexplained part in the upper half of the unconditional net wealth distribution for both first- and second-generation migrants may be due to omitted variables or discrimination. Note, however, that a number of previous studies also find that a relatively small part of the migrant wealth gap is explained (Zhang 2003; Bauer et al. 2011). Furthermore, a drawback of the RIF regression is that we are only able to include data which are available for both groups. Unfortunately, this precludes us from incorporating the time since arrival into our analysis for which the descriptive results in Sect. 4 suggested some relevance, which per definition does not exist for second-generation migrants nor natives. For this reason, we are not able to distinguish cohort effects beyond the indicative analysis presented here. Furthermore, the number of observations prevents us from delving into a more fine-grained analysis of sub-groups. Again, the descriptive evidence was in line with the previous literature in indicating that migrants’ region of origin likely plays a role here (Fig. 5).

Nonetheless, our findings provide a basis for some interesting conclusions: First, the migrant wealth gap for first- and second-generation migrants documented in Sect. 4 is confirmed by the multivariate analysis in this section, especially for the upper half of the distribution. Second, we find evidence of either progressive integration, or a cohort effect—second-generation migrants’ characteristics and net wealth position are much closer to natives’ than to first-generation migrants’. However, the remaining gap cannot be fully explained by the covariates for which we are able to control. This is particularly the case for the upper middle of the distribution, where housing is a highly relevant asset (Fessler et al. 2016), but not for the very top of the unconditional net wealth distribution, where business wealth becomes particularly salient (Rehm and Schnetzer 2015).

Second, these findings may thus suggest that migrants have difficulties catching up in acquiring housing, while there may be fewer differences to the native population in them becoming entrepreneurs. For second-generation migrants, the covariates suggest a *negative* explained wealth gap with respect to natives especially at the top, rather than the very similar raw net wealth data which we observe. For first-generation migrants, the explained gap *exceeds* the raw gap at the top. This indicates that at the top of the distribution, second-generation migrants would be expected to have higher wealth than natives given their characteristics, while first-generation migrants have a lower gap than we would expect from their characteristics. There may thus be some discrimination of second-generation migrants taking place, while wealthy first-generation migrants might be in a favorable wealth situation.

Third, the main socio-demographic factors contributing to explaining the migrant wealth gap are age, marital status, children, and inheritances. Differences in transfers play the most important role in explaining differences in wealth between natives and both first- and second-generation migrants. This suggests that the composition of the migration population, and in particular migrants’ family histories, continue to affect their relative wealth position.

## Robustness Checks

**Fig. 7:**
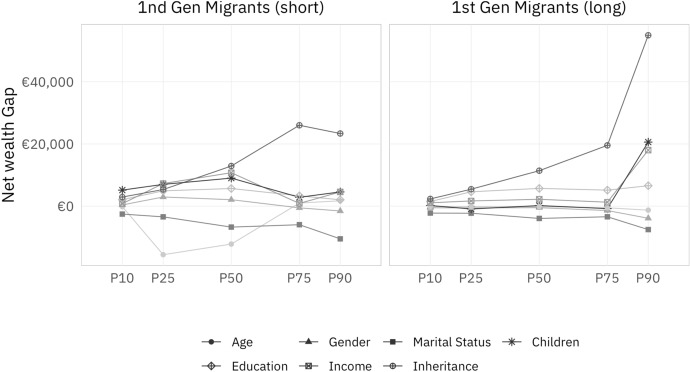
Partial effects of controls for 1st-generation migrants by time since arrival. *Source*: own calculations, HFCS (2014). Note: This figure shows the partial effects of the control variables age, gender, marital status, children, education, income, and inheritance in explaining the migrant wealth gap between natives and two cohorts of first-generation migrants across the unconditional net wealth distribution. Migrants are distinguished between a short ($\leqslant$20 years) and long (>20 years) time since arrival

We check the robustness of our findings by, first, replicating our individual-level analysis at the household level for both net and gross wealth. Second, we replace age with years of work experience, which is arguably a better proxy for income earning capacity, but used less commonly in the literature (Fig. 7).

**Fig. 8:**
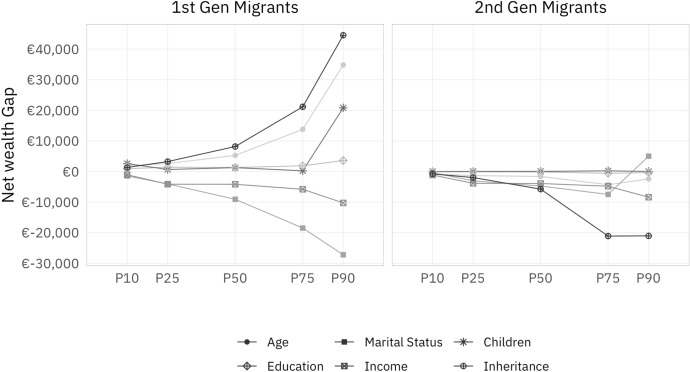
Partial effects of controls at household level, net wealth. *Source*: own calculations, HFCS (2014). Note: This figure shows the partial effects of the control variables at the household level in explaining the migrant wealth gap between natives and first- and second-generation migrants across the unconditional net wealth distribution

**Fig. 9:**
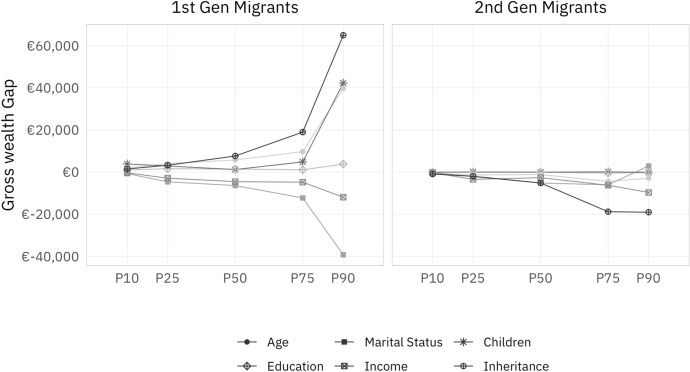
Partial effects of controls on household level, gross wealth. *Source*: own calculations, HFCS (2014). Note: This figure shows the partial effects of the control variables on the household level in explaining the migrant wealth gap between natives and $1{\mathrm{st}}-$ and $2{\mathrm{nd}}$-generation migrants across the unconditional gross wealth distribution

Figure 8 in the Appendix shows that the partial effects of the characteristics explaining the migrant wealth gap transfer very well from the individual to the household level. For first-generation migrants, inheritances remain the single most powerful explanatory variable, followed by age and—again with a negative sign—marital status. The effects are also extraordinarily robust for second-generation migrants; inheritances also explain about €20,000 of the migrant wealth gap at the household level. The same is true for gross wealth (see Fig. 9 in the Appendix).

**Fig. 10:**
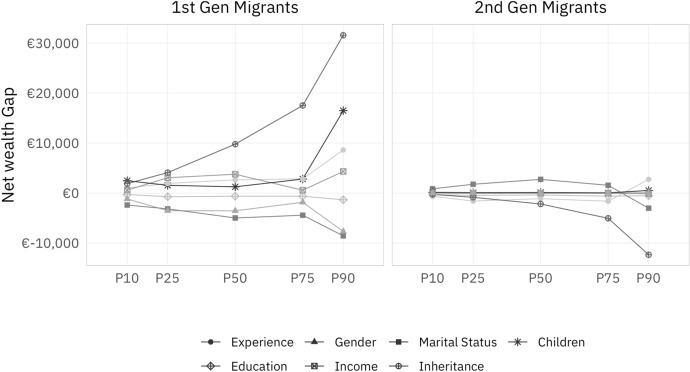
Partial effects of controls with work experience. *Source*: own calculations, HFCS (2014). Note: This figure shows the coefficients of the control variables work experience, gender, marital status, children, education, income, and inheritance in explaining the migrant wealth gap across the unconditional wealth distribution

For our second robustness check, we use years of work experience instead of age (see Fig. 10 in the Appendix). Work experience should better capture our objective, that is, to measure the ability to accumulate wealth through income-earning capacity, as it also takes temporary absences such as parental leave into account. For first-generation migrants, this leads the explanatory power of children to increase—which is in turn offset by all other explanatory variables, whose effects are reduced in magnitude or even turn negative. The partial effects of second-generation migrants remain largely unchanged.

## Conclusion

This paper asks the question how migrants to Austria have been able to integrate into society as measured by their participation in wealth ownership. Using data from the HFCS 2014 (European Central Bank 2014), we first investigate whether there is a migrant wealth gap, that is, whether migrants own less net wealth than natives, and how the migrant population differs from natives in their socio-demographic characteristics. We then apply RIF regressions to decompose the net wealth gap between natives, as well as first- and second-generation migrants at different points of the net wealth distribution, in order to investigate whether the demographic and socio-economic characteristics age, gender, marital status, children, education, income, and inheritance are able to explain the migrant wealth gap across the unconditional net wealth distribution. Austria’s migration sequence of temporary guest-worker policies, family reunification and refugee migration closely resembles that of other European countries, especially Germany and Switzerland.

The descriptive evidence shows two main findings: First, we confirm a positive migrant wealth gap across most of the net wealth distribution. This gap rises especially in the upper half of the distribution before closing (minimally for first-generation migrants) at the top. Within-group inequality rises monotonically from natives to second-generation migrants, to first-generation migrants with a long time since arrival, and to first-generation migrants with a short time since arrival.

Second, we find evidence consistent with substantial catch-up. Second-generation migrants are much more similar to natives, both in terms of their net wealth and—with the exception of gender—in terms of their socio-demographic characteristics. First-generation migrants, in contrast, have a larger migrant wealth gap and a more unequal within-group distribution of wealth. On average, they are also younger, have more children, lower income, and especially lower inheritances than both second-generation migrants and natives. Within first-generation migrants, those with a short time since arrival, while a small sample, are more likely to be male, younger, and less likely to be from the EU than first-generation migrants on average.

We then use a RIF regression, which allows us to decompose the net wealth gap between migrants and natives at different points of the unconditional distribution of net wealth while being path independent. This distributional multivariate analysis confirms an economically significant migrant wealth gap after controlling for socio-demographic characteristics for first-generation migrants, especially for the upper middle of the distribution, where housing wealth is particularly relevant as an asset category. Second-generation migrants’ raw net wealth gap compared to natives, in contrast, is fully explained by our covariates in the upper middle of the distribution. At the top of the distribution, where business wealth is more salient, socio-demographic characteristics would predict first-generation migrants to have a *lower* migrant wealth gap than is observed in the raw data, while second-generation migrants at the top of the distribution would be expected to have *higher* wealth than natives given their characteristics. We cautiously interpret these findings to indicate that migrants appear to have difficulties catching up in acquiring housing wealth both in the first and second generation; and that they may be more likely to become entrepreneurs than the native population, but that they are less likely to succeed in building up very large business wealth in the long run, again compared to natives.

The decomposition of the migrant wealth gap for first- and second-generation migrants shows additional evidence for progressive integration in terms of net wealth, as inheritances are the main explanatory factor for the migrant wealth gap for both first- and second-generation migrants. This leads us to conclude that the composition of the migration population, and in particular migrants’ heritage, continue to affect their wealth relative to natives. Other relevant factors are in particular age for first-generation migrants, which provides some support for different effects of the life-cycle hypothesis between migrants and natives; marital status for both first- and second-generation migrants; and children, education, and income for first-generation migrants.

These findings are on the surface consistent with cohort effects. That is, it is possible that a previous generation of migrants—guest workers, which played a role in migration regimes in Austria, as in Germany or Switzerland during the 1970s—may have built up wealth and bequeathed it to the current generation of second-generation migrants. Furthermore, our descriptive data may also point to a shift within recent migration, since current first-generation migrants with a short time since arrival are better educated than first-generation migrants on average.

However, due to the cross-sectional nature of our data, as well as the limited number of observations in particular for the migrant subsamples, exploring these avenues further must be remitted to future research. Focusing on the concrete channels leading to differences in inheritances would be particularly interesting in this regard; that is, whether the lower inheritances of first-generation migrants are due to lower average wealth levels in migrants’ countries of origin; or whether first-generation migrants are selected from poorer groups of their countries of origin’s population; or whether they are due to their migration status—i.e., refugees versus work migrants, with negative shocks to the wealth of the former in their countries of origin.

A further limitation concerns the dependent variable; we are only able to investigate net wealth since the available data do not permit disaggregating to specific wealth components, such as real estate, businesses, and financial wealth. In addition, a fruitful topic for future research would be efforts to directly measure factors affecting wealth accumulation—that is, differences in saving rates, rates of return, or rates of capital appreciation between migrants and natives—, which may yield clearer insights into the dynamics driving the migrant wealth gap. Scrutinizing differences in saving rates, however, would require better consumption data than is currently available in HFCS. Finally, the factors driving the differences in returns to characteristics (i.e., the structure effect) may be interesting for understanding how discrimination might shape the possibilities for integration. More detailed qualitative research might be especially useful in this regard.

## A Appendix

See Figs. 5, 6, 7, 8, 9, 10 and Tables 2, 3, 4.

**Table 2:** Results of the RIF regression for migrants *Source*: own calculations, HFCS (2014)

	p10	p25	p50	p75	p90
(a) $1^{\mathrm{st}}$-generation migrants					
Intercept	$$-21,964$$	$$-48,589^{**}$$	$$-40,916^{**}$$	11,701	$$46,201^{\dagger }$$
	(17,758)	(15,738)	(14,977)	(11,748)	(27,469)
Age: old	7,826	-3,601	16,834	$26,204^{\dagger }$	$101,363^{*}$
	(9814)	(12,678)	(14,249)	(15,548)	(43,183)
Gender: male	$$-9,207$$	$$-27,952^{**}$$	$$-27,977^{*}$$	-13,049	$$-52,345^{\dagger }$$
	(6638)	(10,313)	(12,266)	(11,756)	(30,900)
Marital St.: single	$$-26,691^{*}$$	$$-36,034^{*}$$	$$-56,088^{**}$$	$$-48,326^{**}$$	$$-84,195^{*}$$
	(12,275)	(15,467)	(16,535)	(14,857)	(42,359)
No children	12,213	15,119	4,503	8,195	46,460
	(12,373)	(15,010)	(17,715)	(18,660)	(43,997)
Education: high	21,231	$$52,898^{**}$$	$$45,611^{**}$$	$$44,137^{**}$$	$$84,914^{**}$$
	(16,269)	(16,426)	(16,350)	(13,848)	(28,666)
Income: high	9,180	$$44,098^{**}$$	$$54,300^{**}$$	10,440	59,353
	(7793)	(12,059)	(14,184)	(14,860)	(37,142)
Inheritance: high	$$20,343^{*}$$	$$46,744^{**}$$	$$109,337^{**}$$	$$190,920^{**}$$	$$302,386^{**}$$
	(7661)	(10,814)	(12,493)	(16,540)	(89,797)
$R^2$	.05	.15	.23	.30	.23
Observations	417	417	417	417	417
(b) $2^{\mathrm{nd}}$-generation migrants					
Intercept	$$-44,506^{*}$$	$$-69,871^{**}$$	$$-44,759^{*}$$	$$-3901$$	$$-17,359$$
	(18,825)	(21,314)	(20,642)	(44,113)	(117,184)
Age: old	$20,366^{*}$	$36,780^{*}$	$35,908^{*}$	$66,098^{\dagger }$	59,530
	(9974)	(14,772)	(17,184)	(36,018)	(96,683)
Gender: male	$-5738$	$-1565$	6800	$-974$	$-44,853$
	(7966)	(11,272)	(12,235)	(30,754)	(78,026)
Marital St.: single	$$-14,603$$	$$-31,036^{*}$$	$$-50,271^{**}$$	-30,649	83,495
	(9412)	(13,017)	(15,041)	(31,359)	(87,643)
No children	$-15,745$	$-3900$	$-4452$	16,907	$-30,506$
	(14,268)	(18,533)	(20,437)	(56,477)	(92,784)
Education: high	$25,751^{*}$	$36,392^{*}$	$32,125^{*}$	$62,654^{\dagger }$	65,044
	(12,795)	(17,096)	(14,783)	(34,054)	(71,736)
Income: high	14,795	$$49,085^{**}$$	$$40,204^{**}$$	49,835	124,152
	(11,275)	(12,738)	(12,345)	(33,506)	(86,343)
Inheritance: high	$$12,571^{\dagger }$$	$$43,575^{**}$$	$$114,747^{**}$$	$$325,429^{**}$$	$$788,258^{**}$$
	(7358)	(10,834)	(13,748)	(44,599)	(178,662)
$R^2$	.09	.20	.34	.31	.24
Observations	337	337	337	337	337

$$^\dagger p<.10$$, $$^{*}p<.05$$, $$^{**}p<.01$$

**Table 3:** Results of the RIF regression for natives *Source*: own calculations, HFCS (2014)

	p10	p25	p50	p75	p90
Intercept	$$-31,390^{**}$$	$$-37,856^{**}$$	7293	$$52,703^{**}$$	$$73,755^{**}$$
	(5860)	(7491)	(9213)	(15,340)	(27,894)
Age: old	$$16,417^{**}$$	$$33,987^{**}$$	$$62,232^{**}$$	$$91,177^{**}$$	$$108,909^{**}$$
	(3657)	(4356)	(6005)	(11,643)	(26,041)
Gender: male	$$-11,710^{**}$$	$$-15,166^{**}$$	$$-14,013^{**}$$	$$-26,787^{**}$$	$$-45,147^{**}$$
	(2558)	(2939)	(3570)	(6985)	(16,589)
Marital St.: single	$$-15,724^{**}$$	$$-41,490^{**}$$	$$-49,850^{**}$$	$$-23,669^{*}$$	$$53,250^{*}$$
	(4774)	(4828)	(5463)	(10,902)	(25,818)
No children	-210	-6174	$$-32,508^{**}$$	$$-40,663^{**}$$	-30,331
	(4761)	(5588)	(7036)	(14,222)	(32,047)
Education: high	$$20,184^{**}$$	$$22,504^{**}$$	$$24,950^{**}$$	$$54,161^{**}$$	$$102,852^{**}$$
	(5004)	(6029)	(7422)	(12,571)	(21,925)
Income: high	$$24,804^{**}$$	$$46,656^{**}$$	$$40,654^{**}$$	$$75,125^{**}$$	$$117,949^{**}$$
	(3346)	(4188)	(5259)	(10,937)	(23,946)
Inheritance: high	$$23,871^{**}$$	$$58,416^{**}$$	$$120,060^{**}$$	$$242,927^{**}$$	$$365,149^{**}$$
	(2407)	(3333)	(5909)	(19,162)	(43,368)
$R^2$	.07	.17	.26	.22	.11
Observations	3416	3416	3416	3416	3416

$$^\dagger p<.10$$, $$^{*}p<.05$$, $$^{**}p<.01$$

**Table 4:** Results of the RIF regression for $1^{\mathrm{st}}$-generation migrants *Source*: own calculations, HFCS (2014)

	p10	p25	p50	p75	p90
(a) $1^{\mathrm{st}}$-generation migrants - short					
Intercept	$$-22,105$$	$$-43,176^{\dagger }$$	$$-60,281^{**}$$	11,500	$$100,991^{**}$$
	(27,038)	(25,074)	(22,031)	(17,116)	(26,727)
Age: old	$-367$	$-38,916$	$-30,413$	2490	4309
	(16,609)	(27,748)	(30,163)	(28,822)	(49,057)
Gender: male	$-3796$	$-28,948^{\dagger }$	$-20,427$	5287	15,076
	(10,781)	(15,963)	(18,345)	(17,050)	(23,674)
Marital St.: single	$-19,568$	$-26,382$	$-51,640^{*}$	$-45,706^{*}$	$-80,677^{*}$
	(18,783)	(24,239)	(23,419)	(20,934)	(31,124)
No children	18,357	24,958	32,103	10,166	16,294
	(18,293)	(20,494)	(22,209)	(23,284)	(36,660)
Education: high	19,015	$44,968^{\dagger }$	$51,725^{*}$	30,360	18,732
	(26,500)	(26,585)	(25,928)	(20,473)	(31,893)
Income: high	4986	$$45,154^{*}$$	$$66,757^{**}$$	5468	28,484
	(13,381)	(17,693)	(19,200)	(19,964)	(32,444)
Inheritance: high	21,400	$$38,972^{*}$$	$$94,149^{**}$$	$$189,776^{**}$$	170,478
	(15,866)	(17,600)	(20,544)	(28,948)	(102,401)
$R^2$	.04	.14	.23	.26	.16
Observations	206	206	206	206	206
(b) $1^{\mathrm{st}}$-generation migrants - long					
Intercept	$-18,437$	$-42,181^{\dagger }$	$-47,144^{*}$	8,315	$-139,152^{\dagger }$
	(22,366)	(23,795)	(19,653)	(15,966)	(77,460)
Age: old	18,660	15,065	18,694	$$60,953^{**}$$	$$147,527^{*}$$
	(14,562)	(23,031)	(21,767)	(21,146)	(72,097)
Gender: male	$$-20,524^{*}$$	$$-21,630$$	-19,003	$$-54,952^{**}$$	$$-152,402^{\dagger }$$
	(8689)	(13,526)	(16,201)	(20,467)	(82,113)
Marital St.: single	$$-35,888^{*}$$	$$-36,650^{\dagger }$$	$$-63,807^{**}$$	$$-55,113^{**}$$	-12,1685
	(16,064)	(22,133)	(20,197)	(20,692)	(107,630)
No children	$$-2241$$	9638	$$-2045$$	8466	$$-222,106^{**}$$
	(15,925)	(25,283)	(24,220)	(23,648)	(82,820)
Education: high	18,279	$$56,191^{**}$$	$$69,420^{**}$$	$$62,605^{**}$$	79,865
	(16,422)	(21,054)	(19,474)	(22,370)	(62,842)
Income: high	17,678	$27,559^{\dagger }$	$36,466^{*}$	20,885	$296,229^{*}$
	(12,521)	(15,139)	(18,266)	(27,633)	(113,586)
Inheritance: high	$$22,974^{*}$$	$$53,711^{**}$$	$$112,365^{**}$$	$$192,533^{**}$$	$$541,285^{*}$$
	(9,336)	(12,352)	(14,674)	(24,104)	(219,788)
$R^2$	.07	.15	.24	.38	.34
Observations	211	211	211	211	211

$$^\dagger p<.10$$, $$^{*}p<.05$$, $$^{**}p<.01$$

## Footnotes

1 The relatively small number of observations for migrants limits the possibilities for fine-grained analysis. For instance, there are only 337 second-generation migrants, which does not allow much heterogeneity in the regression approach. We thus follow Firpo et al. (2018), and transform our socio-economic information into dummy variables. Of course, this also guards against issues of data quality, since this approach is robust to outliers.

2 For a robustness check using years of work experience, which arguably better capture earnings capacity, see Sect. 7.

3 The conservative assumption is thus that inheritances retained their nominal value.

4 The share of imputed observations is well below 1 percent for controls with the exception of inheritances, which are difficult to survey. Income and net wealth have high shares of imputed observations due to their composite nature.

5 Average household size for natives is 2.19, for first-generation migrants 2.59, and for second-generation migrants 2.12.

6 For instance, in the results section below we calculate the RIF at the $10^{\mathrm{th}}$, $25^{\mathrm{th}}$, $50^{\mathrm{th}}$, $75^{\mathrm{th}}$ and $90^{\mathrm{th}}$ percentile.

7 Table 4 shows these results for first generation migrants with a short and long time since arrival.

8 This may be due to us not restricting the sample to working-age adults, and thus including older women, who may have already inherited from their late husbands.
